# Shaping tomorrow: how the STEP training course pioneered noninvasive brain stimulation training for psychiatry in France

**DOI:** 10.3389/fpsyt.2024.1450351

**Published:** 2024-11-25

**Authors:** Marine Mondino, Cécilia Neige, Jean-Marie Batail, Noomane Bouaziz, Maxime Bubrovszky, Samuel Bulteau, Anastasia Demina, Ludovic C. Dormegny-Jeanjean, Ghina Harika-Germaneau, Dominique Januel, Charles Laidi, Virginie Moulier, Marion Plaze, Arnaud Pouchon, Emmanuel Poulet, Maud Rothärmel, Anne Sauvaget, Antoine Yrondi, David Szekely, Jerome Brunelin

**Affiliations:** ^1^ French Society for Biological Psychiatry and Neuropsychopharmacology, STEP Section (Stimulation Transcrânienne En Psychiatrie), Saint-Germain-en-Laye, France; ^2^ Le Vinatier, Psychiatrie Universitaire Lyon Métropole, Bron, France; ^3^ Université Claude Bernard Lyon 1, Centre National de la Recherche Scientifique, Institut National de la Santé et de la Recherche Médicale, Centre de Recherche en Neurosciences de Lyon U1028 UMR5292, PSYR2, Bron, France; ^4^ Centre Hospitalier Guillaume Régnier, Rennes, France; ^5^ Centre d’Investigation Clinique de Rennes - CIC 1414 Inserm, “Neuropsychiatrie du Comportement et du Développement”, CHU Rennes, Rennes, France; ^6^ Université de Rennes, Rennes, France; ^7^ EPS Ville Evrard, Pôle 93G03, Centre de Recherche Clinique, Neuilly-sur-Marne, France; ^8^ La Fondation FondaMental, Créteil, France; ^9^ Pôle de Psychiatrie, Assistance Publique-Hôpitaux de Paris, DMU IMPACT, Hôpitaux Universitaires Mondor, Créteil, France; ^10^ EPSM de l’agglomération lilloise BP4, Saint-Andre Lez Lille, France; ^11^ Nantes Université, CHU Nantes, Movement - Interactions - Performance, MIP, UR 4334, Nantes, France; ^12^ Service Hospitalo-Universitaire d’addictologie, CHU Dijon Bourgogne, Dijon, France; ^13^ Centre de NeuroModulation Non-Invasive de Strasbourg, Hôpitaux Universitaires de Strasbourg, Strasbourg, France; ^14^ UMR CNRS 7357 ICUBE, Université de Strasbourg, Strasbourg, France; ^15^ Centre Hospitalier Henri Laborit, Unité de Recherche Clinique Pierre Deniker, Poitiers, France; ^16^ Centre de Recherches sur la Cognition et l’Apprentissage, Centre National de la Recherche Scientifique (CNRS 7295), Université de Poitiers, Poitiers, France; ^17^ Service Hospitalo-Universitaire de Psychiatrie, Centre d’Excellence Thérapeutique - Institut de Psychiatrie, Centre Hospitalier du Rouvray, Sotteville-lès-Rouen, France; ^18^ GHU Paris Psychiatrie et Neurosciences, Hôpital Sainte Anne, Université Paris Cité, INSERM U1266, Paris, France; ^19^ Univ Grenoble Alpes, Inserm, U1216, Grenoble Institut Neurosciences, “Brain, Behavior and Neuromodulation” Team, CHU Grenoble Alpes; Brain Stimulation Treatment Unit, Grenoble, France; ^20^ Service de Psychiatrie et de Psychologie Médicale de l’adulte, CHU de Toulouse, Hôpital Purpan, ToNIC Toulouse NeuroImaging Center, Université de Toulouse, INSERM, UPS, Toulouse, France; ^21^ Centre Hospitalier Princesse Grace, Unité Neuromodulation, Service de Psychiatrie, Monaco, Monaco

**Keywords:** rTMS (repetitive Transcranial Magnetic Stimulation), transcranial electric stimulation, psychiatric disorders, training course content, education-active learning, neuromodulation, medical education

## Abstract

Over the past three decades, non-invasive brain stimulation (NIBS) techniques have gained worldwide attention and demonstrated therapeutic potential in various medical fields, particularly psychiatry. The emergence of these novel techniques has led to an increased need for robust training programs to provide practitioners, whether clinicians or scientists, with the necessary skills and knowledge. In response, a comprehensive training curriculum for NIBS in psychiatry has been developed in France. This curriculum was developed by a group of researchers and psychiatrists interested in the clinical application of NIBS in psychiatry, called STEP - Stimulation Transcranienne en Psychiatrie, under the auspices of the French Association of Biological Psychiatry. This perspective outlines the development and implementation of this course, tracing its inception, the evolution of the program, and the challenges encountered along the way. The position of the course in the national and international environment and its future prospects are also discussed. Through this perspective, we aim to summarize the collaborative efforts to promote NIBS teaching and research in French psychiatry.

## 1 Introduction

Noninvasive brain stimulation (NIBS) encompasses a range of techniques that allow the modulation of brain activity and neural circuits *in vivo*, without the need for surgery or anesthesia. This is achieved either through the application of an electromagnetic field, as is the case with transcranial magnetic stimulation (TMS), or through the application of weak electrical currents to the scalp, as is the case with transcranial electrical stimulation (tES) including transcranial Direct Current Stimulation (tDCS). The non-invasive nature of these methods, coupled with their ability to target specific brain regions, have made them appealing for both research and clinical applications. NIBS approaches have demonstrated therapeutic potential across a range of neuropsychiatric conditions, including pain, stroke, tinnitus, dementia, depression, schizophrenia, substance use disorder, and anxiety disorders (see 1, 2 for reviews and clinical guidelines).

As these techniques become more widely applied in clinical practice, it is essential for NIBS practitioners to receive specialized training and acquire technical expertise to ensure their safe and effective administration, given the inherent complexity of these techniques. In response to this need, NIBS training programs have emerged across the world to ensure practitioners are adequately prepared. In this perspective, we aim to describe the curriculum development and the implementation of a training course on the use of NIBS in psychiatry in France. Given the lack of specific academic training in noninvasive brain stimulation in France, the course was initially developed by a group of psychiatrists and neuroscientists interested in the clinical application of TMS to patients with psychiatric disorders. First, a brief historical overview will outline the path that led to the convening of an inaugural meeting to define common objectives and to form the association central to the genesis of the first TMS training program. The content of the courses, their organization, and their evolution over the years will be then described.

## 2 Once upon a time in France

**Figure 1:**
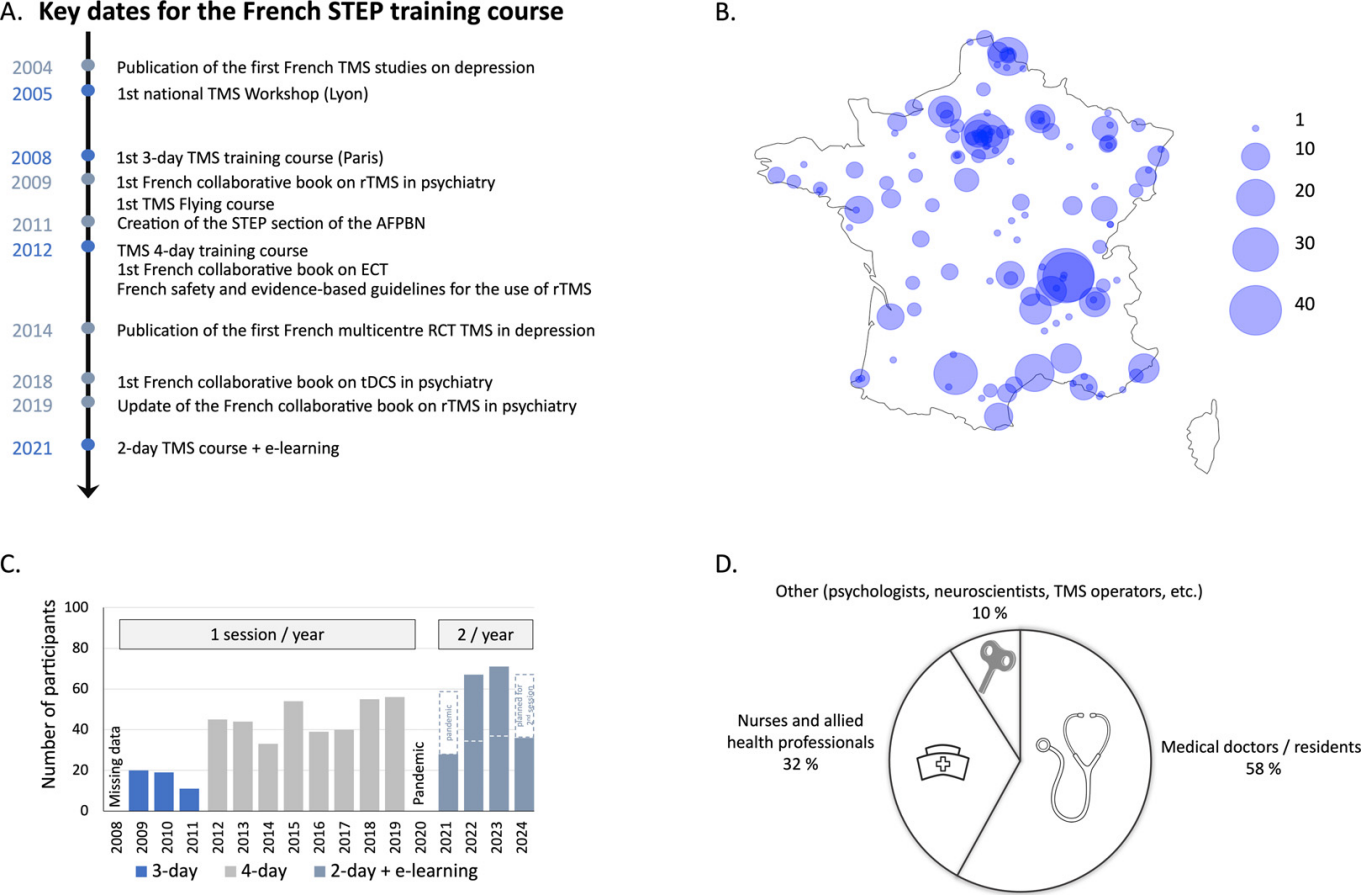
STEP training courses illustrated. **(A)** Timeline of key dates for the French STEP training courses (blue) and key French publications (grey-blue). **(B)** Geographical distribution of participants by city in metropolitan France and Corsica. **(C)** Evolution of the number of registrations and course formulas over the years. The number of registrations includes only regular courses and does not include flying modules. **(D)** Professional profiles of the participants in the STEP training courses.

In the early 2000s, some original articles investigating the clinical benefits of TMS in patients with various psychiatric disorders were published in international peer-reviewed journals by different groups in France who did not know each other. In 2004, three publications with a limited sample size and different stimulation parameters were published by three different groups investigating the interest of TMS in patients with major depressive disorder (**Figure 1**). They came from the Sainte-Anne Psychiatric Hospital and the University of Paris (3), Le Vinatier Psychiatric Hospital and the University of Lyon (4), and the EPS Ville-Evrard Psychiatric Hospital in Neuilly-sur-Marne (5). In addition to depression, some authors from the same groups also published promising results in patients with mania (6) and in patients with resistant auditory hallucinations in schizophrenia (7).

In order to facilitate collaboration between the actors in this emerging field, we decided to organize a first national meeting with all those interested in the use of TMS for psychiatric purposes, whether medical, paramedical, or neuroscientist. Representatives from several French cities (Besançon, Bordeaux, Lyon, Paris, Clermont-Ferrand, Strasbourg, Lille, Rouen, Créteil, Neuilly-sur-Marne, Grenoble) presented the work being developed in their laboratories. This first workshop took place in Lyon on March 18, 2005 and had 3 objectives:

- Objective 1: Research. To develop a large, multi-center, national randomized controlled trial in the field of depression, according to high research quality standards in order to gain international recognition and visibility.
- Objective 2: Regulatory policies. To create a national database to accelerate the recognition of TMS as a treatment for depression in real-life situations by national health authorities.
- Objective 3: Education. To share support for the organization of an annual national course to standardize application methodology in accordance with good clinical practice and safety guidelines.

In order to achieve our objectives, in July 2006 we created a French association under the law of 1901 called “*Club rTMS & psychiatry*”. In 2010, the scope of the association was extended from exclusively TMS to all non-invasive electrical and magnetic stimulation in psychiatry (including electroconvulsive therapy (ECT) and low-intensity tES), and the association was incorporated as a subsection of the French Association of Biological Psychiatry and Neuropsychopharmacology (AFPBN), called STEP (“*Stimulation Transcrânienne En Psychiatrie*”, website available at: https://step-afpbn.org/accueil).

Regarding objective 1, we obtained national funding (national PHRC 2007 - DGOS) to develop a multicenter study involving 170 patients with treatment-resistant major depression from 18 centers across the country (8). Regarding objective 2, some research centers started to share data on patients receiving TMS as an off-label treatment and published one of the largest cohorts of patients with depression receiving TMS evaluated with a standardized hetero-questionnaire (9). Finally, in accordance with objective 3, we created the annual STEP course on non-invasive brain stimulation in psychiatry, with the aim of homogenizing practice in terms of good clinical practice and safety guidelines, increasing collaboration and training TMS practitioners, doctors, nurses, psychologists, and neuroscientists interested in using NIBS for psychiatric purposes.

## 3 The first French TMS training course

The first French TMS training course was scheduled in 2008. We proposed a 3-day course with 3 training modules (one module per month, from April to June). It was held at the EPS Ville-Evrard Psychiatric Hospital in Neuilly-sur-Marne (F-93330), near Paris. The first training module was a one-day module open to all TMS practitioners and people who were about to be equipped with a TMS stimulator. The educational objective was to provide sufficient information on the safe clinical use of TMS in psychiatric patients. It covered the history of the technique, its safe use and contraindications, and an overview of recent results from the literature regarding clinical application (mainly in depression and schizophrenia). Participants were trained to use the TMS device to assess the resting Motor Threshold (rMT) (2-hour sessions) and to install the participants and select the appropriate and accurate parameters of stimulation depending on the clinical situation (2-hour sessions, including target localization) and the available TMS device. The second module was designed for more advanced users. It provided theoretical background on the potential mechanisms of action of TMS, how to measure cortical excitability with single and paired-pulse TMS, and the application of such measures in patients with psychiatric conditions. Participants were then invited to practice single and paired-pulse TMS and learn to assess common measures of excitability such as cortical silent period (CSP), short-interval intracortical inhibition (SICI), intracortical facilitation (ICF), and long-interval intracortical inhibition (LICI), with the help of a neurophysiologist. The third module, also aimed at more advanced clinical users and researchers, provided theoretical background on the interest of combining magnetic resonance imaging (MRI) and functional MRI (fMRI) for accurate targeting of cortical regions of interest using neuronavigation systems. Participants were invited to practice with the neuronavigation systems available on site.

## 4 The cruise phase, from 3 to 4 training modules

Since the first TMS training course, we have kept the idea of offering a course over several days, with the level of difficulty and proficiency increasing day by day, but for practical reasons, we chose to group the different modules on consecutive days. Registration for the modules was independent and “à la carte”: participants could choose to register for only one of the modules. They could take all 3 modules at once, or take Module 1 in one year and return in subsequent years to complete the training with the other modules. This formula was reinforced by participant feedback collected through anonymous questionnaires after each session, which identified two distinct needs: staff who would not prescribe but only operate the stimulation in clinical practice felt that Modules 2 and 3 were too advanced and not directly relevant to their practice, while some physicians, researchers, and more advanced users asked for additional in-depth scientific content. We followed the 3-day formula for 4 years (2008 to 2011) and then, since 2012, we moved to an annual 4-day training course, always held at the end of September in Lyon, at Le Vinatier Psychiatric Hospital. This formula was expanded to encompass additional NIBS techniques, including electroconvulsive therapy (ECT) and transcranial electrical stimulation, in response to the growing demand for these techniques. The first two days (Module 1&2) were still dedicated to TMS. The third day (Module 3) was devoted to other neuromodulation techniques, in particular ECT and tDCS, with theoretical courses on general principles and mechanisms of action, clinical applications, and hands-on sessions for each technique. Finally, the last day, the “scientific day” (Module 4) was focused on the cognitive, biological, and behavioral effects of the techniques, with more in-depth points on the mechanisms of action of rTMS, cortical excitability, the contribution of imaging in neuromodulation treatments, and the contribution of animal models. The pedagogical content of the course has been partially modified each year from 2012 to 2019 in order to provide participants with the most up-to-date training.

## 5 Towards the actual formula combining an onsite 2-day STEP training course with e-learning

After a year and a half off due to the COVID-19 pandemic, from 2020 to summer 2021, we redesigned the program and resumed with a new format in autumn 2021. The new training course program combines a two-day on-site course in Lyon with over 25 files of e-learning sessions. The introduction of this new 2-day format has enabled us to respond to the growing demand for training by increasing the frequency of the training courses to two sessions per year, one in spring and one in autumn, starting in 2022. This change was essential to address the growing waiting list, particularly after the course cancellations during the pandemic, as organizing two sessions of four days would have been logistically impractical.

The following section will provide a detailed description of this updated training course. This training course follows the recommendations of the International Federation of Clinical Neurophysiology (IFCN) (10) and gives special emphasis on presenting the most up-to-date recommendation in terms of both safety (11) and evidence-based guidelines for the clinical use of both tES and rTMS. Our educational content is designed to include several core elements: knowledge of how to screen patients for contraindication criteria for the different techniques, adherence to safety guidelines, understanding of technique-specific parameters and their safe application, the use of standardized measures to assess clinical effects, and the systematic assessment of potential adverse effects. The e-learning sessions cover the dimensions of the history of the technology, mechanisms of action (ECT, TMS, and tES, neurophysiological evidence), evidence-based guidelines (in particular the latest evidence in all psychiatric and addictive conditions), ethical considerations, titration (ECT and TMS), targeting, in-home application, safety recommendations, recent advances, optimal parameters, the potential benefits of robots, and other related topics (see **Table 1** for more details). The e-learning platform was accessible to all participants before the on-site course and comprised videos, annotated slideshows, and original research articles from the international literature (mainly evidence-based guidelines, and safety recommendations including (1, 2, 11–17) that are freely available for students on the website platform for students). Some courses are mandatory to complete in order to validate the training, while others are freely accessible for participants interested in learning more. This approach allows us to meet the expectations of participants at different levels of need and advancement. As the on-site training is particularly dedicated to practical skills in the field of psychiatry and mental health, more theoretical lectures on neurophysiological, neurological and motor (including CSP, LICI, SICI, ICF, which have no clear direct clinical application in psychiatry, (15) effects of NIBS are available online. The on-site modules combined lectures (morning) and practical sessions (afternoon) dedicated to clinical application in psychiatry. In general, the morning of the first day provided an overview of safety guidelines and the latest evidence-based indications for NIBS in psychiatric disorders (mood disorders, schizophrenia, anxiety disorders, substance use disorders), the place of all NIBS techniques in the therapeutic algorithm (tDCS, TMS, deep TMS, ECT, pharmacology, psychotherapy). In the afternoon, a training session dealt with how to inform and obtain written consent for TMS, how to screen for TMS contraindications, how to install and monitor a rTMS session with a patient (e.g., screening for potential side effects), and how to program the device with appropriate stimulation parameters. Participants were also trained in the basic application of rTMS: proper coil handling and placement (location, orientation, angulation), methods for locating cortical targets (e.g., 10-20 EEG international system, Beam F3 (18), neuro-cardiac guided TMS (19), neuronavigation system) and selection of stimulation parameters; finding the motor hotspot, rMT determination using electromyographic measures and/or visible twitch. Basic neuroanatomical principles were covered to facilitate the location of the main targets in psychiatry including the right and left dorsolateral prefrontal cortex (DLPFC), the left temporoparietal cortex, the orbitofrontal cortex, the pre-supplementary motor area. On the second day, we provided an overview of the mechanisms of action of tES and ECT, and how to implement a NIBS unit in France (20). In the afternoon, hands-on sessions were organized to learn how to perform a tES session and titration of ECT according to safety guidelines and good clinical practice. For instance, for tES it includes electrodes placement, setting stimulation parameters (e.g., intensity, frequency, duration, ramp up/down), impedance check, and assessing the potential side effects (with a standardized questionnaire, see (16). A final session consisted of the application of a clinical situation, and how to inform and obtain consent. In 2024, during the on-site sessions, we decided to provide an overview and comparison of rTMS and tDCS for all the main indications in psychiatry, but also outside the field of psychiatry (see the full program in **Table 1**).

Table 1Curriculum of the 2024 French STEP 2-day onsite course.

**Table:** 

DAY 1	
08h30-08h45	Attendee welcome
08h45-09h00	Introduction to the STEP training course
09h00-10h15	rTMS & tDCS for mood disorders: from evidence-based guidelines to applications in the clinical settings
10h15-11h00	rTMS & tDCS for schizophrenia dimensions
	*Break – Booth visit*
11h30-13h00	rTMS & tES mechanism of action and indication outside of the field of psychiatry
	*Break – Booth visit*
14h00-17h00	Three 45-min practical sessions (groups of 10 max)
Session 1	How to start and monitor a TMS session? Safety guidelines and reglementary consideration Screening for eligibility, the process for obtaining written informed consent, and screening for TMS contraindications
Session 2	Target detection using the 10/20 system and Beam F3 (and F4) Proper coil handling and placement (location, orientation, angulation) Finding the motor hotspot, rMT determination using EMG and/or visible twitch, DLPFC Location (and other target)
Session 3	Neuronavigation systems - theoretical and practical aspects

**Table:** 

DAY 2	
08h45-09h00	Attendee welcome
09h00-10h00	ECT positioning and application
10h00-10h45	rTMS & tDCS for anxiety-related disorders: from evidence-based guidelines to applications in the clinical settings
	*Break – Booth visit*
11h15-11h45	rTMS & tDCS for substance use disorders: from evidence-based guidelines to applications in the clinical settings
11h45-12h15	The place of deep- rTMS
12h15-12h45	How to implement a NIBS Unit
	*Break – Booth visit*
13h30-16-30	Three 45-min practical sessions (groups of 10 max)
Session 1	How to start and monitor a tES session? Safety guidelines and reglementary consideration Material description, electrodes placement, parameters settings, impedance check, side effect assessment
Session 2	Which NIBS in which situation? Practical cases and place of other interventional psychiatry tools (Ketamine, psilocybin, etc.) and other brain stimulation approach (MST, DBS, etc.),
Session 3	Discussion time, evaluation of the formation by students feedback

Finally, to improve the quality and quantity of participant feedback, we have introduced an on-site feedback session from October 2024, where participants will scan a QR code at the end of the training to complete a survey, with the aim of obtaining more comprehensive and reliable responses for continuous course improvement.

## 6 The flying STEP TMS course

In order to be as close as possible to TMS practitioners, and because it is not always easy to find financial support to travel for several days to attend a TMS training course, we have proposed a Flying STEP TMS course consisting of a one-day training that can be held anywhere in France. This course is particularly suitable for units where rTMS clinical activity is about to begin, enabling direct contact between experienced instructors and the entire future neuromodulation team, including the paramedical members who cannot all have been present at the main training sessions.

The TMS course program is very tight from Module 1 of the 2-day training. It provides in the morning an overview of evidence-based guidelines for the use of TMS in psychiatric conditions, safety, and regulatory recommendations. Hands-on sessions in the afternoon allow to determine the rMT and facilitate the localization of the main cortical target in psychiatry (mainly the right and left DLPFC and the left temporoparietal cortex), to set up and monitor a TMS session, to determine the good parameters in the appropriate indication. This Flying STEP TMS course has been held in several French cities since 2009, including Lyon (2009), Lille (2010), Marseille (2011), Rennes (2012), Nice (2013), Toulouse (2013), Nantes (2014), Strasbourg (2016), Lille (2022), Paris (2023), and also in Monaco (2018).

## 7 STEP training courses in numbers

Since 2009, the STEP training course has registered over 650 subscriptions. In the first three years, with the 3-day format, we typically hosted about 15-20 participants per module. With the transition to the 4-day format, the number of participants per module has rapidly increased to approximately 45-50 in 2019. To make it easier to organize the hands-on sessions with around 10-12 participants and to ensure that everyone is involved and can practice, we have limited the number of participants per module to around 30-35. Confirming the interest in the technique in France and the need for specific training, the maximum number of participants was always reached. A waiting list has been set up to ensure that those who cannot attend will be able to attend the next session. The introduction of the new 2-day format in 2021 has allowed us to increase the frequency of the training courses to twice a year, with one session in the spring and one in the autumn.

The vast majority of participants (95%) came from 140 different cities in France, covering the metropolitan territory (see **Figure 1B**). Only 5% of participants came from other countries, with the majority coming from Switzerland and Monaco, and smaller proportions from Belgium, Morocco, Algeria, Tunisia, Poland, Romania, and Canada. The profile of the participants was as follows: more than half (58%) were medical doctors or residents, a third (32%) were nurses and allied health professionals (e.g., caregivers, radiology technicians), and 10% had other profiles (psychologists, neuroscientists, TMS operators, etc.). This heterogeneity of professional profiles is also found among the teachers, who are psychiatrists, neurophysiologists, addictologists, neuroscientists (researchers, engineers, post-docs), and clinical research nurses. All speakers are involved in brain stimulation research, as evidenced by publications in international peer-reviewed journals. Between five and ten different speakers gave a lecture every day. The duration of each course was usually 30 minutes and never exceeded an hour and a half, in order to maintain participant’s attention. If they agree, after the course, participants are added to a STEP membership list (currently around 300 people), whether or not they are full members of the association, and receive regular newsletters and information about STEP activities, including the Scientific Day or the webinar, to enable them to maintain their knowledge of NIBS.

## 8 Accreditation of the training course

The training course received an accreditation for continuing education by the CNQSP (*Collège national pour la qualité des soins en psychiatrie* - National College for the Quality of Psychiatric Care) in collaboration with the AFPBN (registration number: 53350920735). This agreement includes an evaluation of the knowledge and skills on NIBS acquired during the STEP course and allows participants to be reimbursed by their employer and to receive a certificate of training.

## 9 Promotional strategies

The STEP group, as a section of the AFPBN, benefits from the support of a specialized communication agency and relies on this agency to manage the membership list, organize the training course sessions and other STEP-related events (such as webinars, see section 10.1). They are also responsible for promoting the STEP course through advertisements in newsletters to AFPBN/STEP members, dissemination on the AFPBN and STEP websites, and social networks. STEP instructors also promote the training courses during their teaching activities, webinars, and scientific days, as well as at national and local psychiatry congresses. In most cases, advertising is unnecessary because many hospital teams that provide NIBS therapy are already familiar with us and routinely send their new recruits to our training courses. Additionally, individuals often reach out to us after consulting well-known medical doctors who practice rTMS, and these doctors are either members of STEP or are familiar with our organization. We are also recognized by manufacturers who refer individuals to our training courses.

## 10 Economical model

In order to reduce the cost of the course and decrease the registration fees for all the participants, we have decided to work hand in hand with the manufacturers. This collaboration allows us to reduce the cost of shipping the equipment, which is covered by the manufacturer, and also allows us to have the necessary equipment available for the hands-on sessions. Manufacturers can also pay for a booth to display their equipment during the attendees’ welcome and the breaks. It is also important for the participants to meet different manufacturers, discover the equipment they propose, compare them (either technically or practically for daily use), and discuss with the teachers the pros and cons of buying this or that equipment. This is a win-win-win model (students - manufacturers - teachers). Each year, all manufacturers or distributors of NIBS equipment for the French market are invited to attend and present their equipment, and usually 3 or 4 manufacturers attend the event.

To reduce the cost of the Flying STEP TMS course, it is usually held the day before a scientific meeting organized by the association. This allows the instructors to be on site for 2 days, one day for the course and one day for the scientific meeting.

## 11 Discussion

### 11.1 Other actions to disseminate knowledge on NIBS in psychiatry

The STEP group has also promoted neuromodulation in psychiatry by proposing other educational initiatives. For instance, the group has coordinated and published three books on NIBS techniques: one on rTMS published in 2009 (21) and updated in a new edition in 2019 (22), one on ECT in 2012 (23), and one on tDCS in 2018 (24).

The Group is also offering free webinars at the rate of one every six months from 2022, with an average of 110 participants per online session. The webinars are also available offline on our YouTube & LiveStorm channels. These webinars serve as “master classes” and cover general topics on techniques that may be of interest to the medical community, such as the recent ones on “ECT in everyday life”, or “accelerated rTMS for depression” (in collaboration with the ESBS), and more specific topics at the intersection of other topics, such as the one on “The suicidal brain”, organized in collaboration with the SECS (*Section d’Etudes des Conduites Suicidaires*) section of the AFPBN, which included a presentation on neurostimulation in the treatment of suicidal crisis.

The STEP section also initiates the organization of original symposia at national psychiatry conferences. This is the case of the day of scientific association during the CFP (*Congrès Français de Psychiatrie*). The members of the STEP section also represent the organization during the symposium they propose at national and international conferences of psychiatry, neuropsychopharmacology, or brain stimulation. During these conferences, we also organized hands-on sessions for residents in psychiatry. We developed an alternative pedagogical strategy for these sessions, through two separate 1-hour serious escape games on ECT and rTMS. Participants had one hour to find the right stimulation parameters for a given patient in a given situation.

To promote the use of NIBS in psychiatry in France, some members of the group also regularly published updates and recommendations in French to reach a large non-English-speaking audience in the French-speaking world (25–29).

### 11.2 The current NIBS training situation in France

In addition to the training courses offered by the STEP group, three courses providing a brief overview of some NIBS and their application in psychiatry have recently been made available in France. At least three courses lead to university diplomas: (i) the inter-university diploma in ECT and rTMS, organized and delivered by the Universities of Bordeaux, Lyon 1, and Nantes, (ii) the inter-university diploma in psychopharmacology and cerebral stimulation therapeutics, organized and delivered by the universities of Paris-Sorbonne and Rouen, and iii) the university diploma in therapeutic neuromodulation, organized by the Paris-East Créteil University. Most of the teachers involved in the STEP course are also regular teachers or organizers of the university diplomas. This allows for constructive exchanges and avoids redundancy between courses. The target participants are also quite different from course to course, open or not to people with different initial training (*e.g.*, M.D., Ph.D., nurse, engineer, etc.) and there are also different proportions of theoretical and practical skills to be acquired in each course. Several other local university degrees that integrate NIBS are in preparation across the country.

### 11.3 Future directions

The STEP group is currently exploring various avenues for the development of its training courses. Firstly, in-depth reflection is required on the degree-granting nature of the training course, at the individual level, but also for centers practicing NIBS. In this latter context, the STEP group could propose a national accreditation framework for centers practicing NIBS techniques, specifying best practices in supervision, quality of care provided, safety of stimulated individuals, and data protection, according to international standards in this field (10).

Furthermore, in the context of potential recognition of the training through a diploma or accreditation, the group is seeking to align the educational content of its training courses with those of other European countries through the European Society For Brain Stimulation (https://www.brain-stimulation.eu/) of which some members of the STEP group teaching in the training courses are also members. Nevertheless, it is of the utmost importance to maintain French-language training in order to continue reaching a French-speaking audience.

Another potential avenue for the courses could be the use of novel pedagogical methodologies. To ensure a thorough grasp of the training and its practical application, participants may be asked to compile a portfolio at the conclusion of both the training and supervision sessions. A portfolio is a compilation of documents, work samples, projects, and achievements organized to showcase an individual’s skills, progress, or qualifications in the field of neurostimulation. Portfolios can be presented in various formats, including physical or digital versions. Participants are responsible for selecting and presenting the contents of their portfolio to demonstrate their capabilities and accomplishments.

To make the training more practical and to assess knowledge in an individualized manner, we propose to implement OSCE (Objective Structured Clinical Exams). It allows students to demonstrate their ability to apply theoretical knowledge to real-world situations. OSCE assessments often include practical exercises, simulations, or case studies where students have to identify hazards, propose safety measures, and address safety concerns. This type of assessment will prepare students to react to situations of uncertainty (*e.g.*, choice of stimulation type or setting), as well as emergencies (*e.g.*, epileptic seizure during an rTMS session, status epilepticus during an ECT session - OSCE were already used to discuss epilepsy-related situations (30) or communication difficulties (e.g., with an ECT-skeptical professional or patient - OSCE were also used to improve communication about other stigmatized cares such as vaccines (31). Precise modalities remain to be defined, especially the use of virtual reality (VR) to create immersive environments (*e.g.*, intervention room for ECT) and enhance the number of scenarii without the need to set up multiple fully equipped examination rooms. VR based OSCE already showed promising results in another intervention training in emergency situations (*i.e.*, cardio-pulmonary resuscitation) (for review see 32). Another way to improve practitioners’ skills would be to use simulation-based teaching with the use of mannequins. The technical and practical nature of neurostimulation lends itself well to these innovations with the possibility of varying scenarios in a controlled manner. Furthermore, including expert patients or peer-supporters who have benefited from these techniques would further sensitize practitioners to the best approaches (explanation, organization, delivery) and facilitate destigmatization and acceptance of these treatments by the community.

Finally, the STEP group is committed to obtaining recognition from the French health authorities for the use of NIBS in clinical practice, by launching a petition (https://step-afpbn.org/actualite-29) and alerting the national press (33) and the international scientific community (34). The STEP group also aims to continue its efforts to promote research into the therapeutic effects of NIBS in psychiatry by bringing together teams using these techniques and providing the network and support needed to develop large randomized clinical trials (*e.g.* 35–37). By joining the European Society for Brain Stimulation (ESBS) in 2023, the STEP group will join efforts to promote the safety and clinical interest of NIBS at the European level (38, 39).

Currently, training programs for these techniques vary widely internationally, ranging from a brief lecture during residency to a comprehensive fellowship dedicated to interventional psychiatry. For effective non-invasive brain stimulation (NIBS) application, physicians, licensed psychologists, and technicians must be proficient in the scientific, clinical, and technical principles of the procedure. This includes understanding indications and contraindications, being fully proficient in application of the treatment (dose determination, targeting, session monitoring) (40).

Echoing with the work of STEP section over the last years in terms of training, the specificity and potential of non-invasive brain stimulation techniques (such as rTMS, tDCS, ECT, …), there is a need to standardize training for professionals to ensure both efficacy and safety. France - thanks to STEP structuration - has now a mature training program to develop a dedicated interventional psychiatry which could be defined as a “subspecialty that pertains to the conduct of procedures involving the administration of electrical or other forms of focused energy (*e.g.*, magnetic, sonic) to alter brain network function in a manner that ameliorates symptoms” (41–43). However, interventional psychiatry may also be considered by some to include treatment with psychedelics (e.g., off-label use of ketamine, research protocols with psilocybin) and invasive neuromodulation (such as deep brain stimulation – DBS, and vagal nerve stimulation - VNS) that are not included in the STEP training courses. However, their place in the treatment algorithm is discussed in a practical workshop using clinical case scenarios. Furthermore, techniques like Magnetic Seizure Therapy (MST), transcranial Focused Ultrasound Stimulation (FUS), and transcranial Temporal Interference Stimulation (tTIS) have not yet been integrated to the STEP training courses due to less availability, expertise, and demand in the current French landscape for clinical purposes in psychiatry; however, STEP may consider positioning itself to incorporate these techniques in the future.

In France, there is a pressing need for official NIBS training. For instance, despite the growing number of rTMS centers and available training resources such as university hospitals and scientific communities (*e.g.*, the AFPBN, the French Society of Clinical Neurophysiology), the absence of standardized training and practice regulations poses a risk of inconsistent and suboptimal treatment practices. This lack of regulation could potentially hinder the development and application of rTMS, resulting in missed opportunities for patient care.

## Data Availability

The original contributions presented in the study are included in the article/supplementary material. Further inquiries can be directed to the corresponding author.
